# Prevalence and molecular characterization of *Enterocytozoon bieneusi* in endangered Eld’s deer (*Rucervus eldii*) in Hainan, China

**DOI:** 10.3389/fvets.2025.1521055

**Published:** 2025-01-27

**Authors:** Yun Zhang, Guangxu Ren, Qingqing Lu, Jiaqi Li, Yu Qiang, Youyou Li, Xiuyi Lai, Yuan Wang, Xingyue Yu, Sheng Lei, Yu Li, Yunxing Chang, Xianrong Liu, Xuning Qi, Zhi Xie, Tingting Li, Jiang Du, Rui Duan, Xinyu Chang, Hesheng Wang, Gang Lu

**Affiliations:** ^1^Department of Pathogenic Biology, Hainan Medical University-The University of Hong Kong Joint Laboratory of Tropical Infectious Diseases, Key Laboratory of Tropical Translational Medicine of Ministry of Education, School of Basic Medicine and Life Sciences, Hainan Medical University, Haikou, China; ^2^Department of Tropical Diseases, The Second Affiliated Hospital of Hainan Medical University, Haikou, China; ^3^Department of Infection Control, The First Affiliated Hospital of USTC, Hefei, China; ^4^Department of Nuclear Medicine, The 928th Hospital of PLA Joint Logistics Force, Haikou, China; ^5^Hainan Bangxi Provincial Nature Reserve Administration, Baisha, China; ^6^Bawangling Branch of Hainan Tropical Rainforest National Park Administration, Changjiang, China; ^7^Hainan Datian National Nature Reserve Administration, Dongfang, China

**Keywords:** Eld’s deer, *Enterocytozoon bieneusi*, prevalence, genotype, Hainan Island

## Abstract

**Introduction:**

*Enterocytozoon bieneusi* is one of the most frequent microsporidia species causing digestive disorder mainly diarrhea in humans and animals. Eld’s deer (*Rucervus eldii*) is the class I national key protected wildlife and only distributed on Hainan Island in China. No report on the prevalence and molecular characterization of *E. bieneusi* in wild Eld’s deer worldwide.

**Methods:**

217 fecal samples were collected from Eld’s deer in two isolated habitats of a nature reserve in Hainan, and examined by nested Polymerase Chain Reaction (PCR) targeting the internal transcribed spacer (ITS) region.

**Results and discussion:**

The overall prevalence of *E. bieneusi* in Eld’s deer was 17.5% (38/217), with 13.5% (12/89) and 20.3% (26/128) in habitats 1 and 2, respectively. Seven ITS genotypes were identified, including five known genotypes: D (*n* = 19), Peru11 (*n* = 10), EbpC (*n* = 5), Peru8 (*n* = 1) and Type IV (*n* = 1), and two novel genotypes: HNED-I and HNED-II (one each). Genotypes Peru8 and Peru11 were firstly identified in cervids. Phylogenetic analysis showed that all the detected genotypes belonged to zoonotic Group 1. The results implied that the further research on threaten of *E. bieneusi* to endangered Eld’s deer and potential risks for public health is necessary.

## 1 Introduction

Microsporidia are widely spread obligate intracellular pathogens that infect a broad range of hosts, including both vertebrates, such as humans, and invertebrates (1, 2). There are about 220 genera and 1,700 species of microsporidia, which are classified based on their ultrastructural features, developmental cycle, host–parasite relationship, and molecular analysis (3). Of the 17 microsporidian species known to infect humans, *Enterocytozoon bieneusi* is by far the most frequent species in the clinical setting and generally presents as chronic diarrhea and wasting syndrome, particularly in immunocompromised individuals such as those with AIDS or transplant recipients, as well as travelers, children, and the elderly (4–6). It was transmitted by fecal-oral route, mainly by ingestion of contaminated food and water with spores (7–9). Due to the difficulty of microscopic identification for small size, *E. bieneusi* is mainly detected and genotyped by the method of nested polymerase chain reaction (PCR) targeted internal transcribed spacer (ITS) region and sequence analysis (10). To date, around 900 different genotypes of *E. bieneusi* have been identified and classed into 13 phylogenetic groups (group 1–13) (11). The first two clusters (Groups 1 and 2) accounted for a significant proportion (94%) of the total genotypes, encompassing the majority of known human-pathogenic genotypes and zoonotic genotypes (12). Group 3–13 were host adaptation groups and might be present in specific hosts and wastewater (5, 12).

Eld’s deer (*Rucervus eldii*) is a rare and globally endangered tropical deer species, belonging to Artiodactyla, Family Cervidae and Subfamily Cervinae. It is distributed across Southeast Asia, Southern China and the northeastern part of India. Because of illegal poaching and severe habitat encroachment, the global population of Eld’s deer has sharply declined (13). It has been listed in Appendix I of the Convention on International Trade in Endangered Species of Wild Fauna and Flora (CITES) and classified as endangered on the Red List of Threatened Species by the International Union for Conservation of Nature (IUCN) and the class I national key protected wildlife in China (14–17). In China, Eld’s deer is only distribute in Hainan Island. Due to the rapid destruction of habitats and intense hunting by humans, only 26 individuals was remained in Hainan at end of 1970s (18). Despite fact that the Eld’s deer population has recovered and grown after over 40 years of development and preservation, it continues to be extremely vulnerable to extinction because of inbreeding, poor genetic diversity, the diminishing evolutionary capacity of tiny populations, high population density, and infectious diseases (19). At present, no information about *E. bieneusi* in endangered wild Eld’s deer was reported. The aims of this study were to investigate the prevalence and molecular characterization of *E. bieneusi* in wild Eld’s deer in Hainan, and provide valuable information for development and preservation of this endangered wildlife.

## 2 Materials and methods

### 2.1 Ethics statement

The collection of fecal samples from Eld’s deer have been permitted by Hainan Bangxi Provincial Nature Reserve without human disturbance to the animals. The non-invasive sampling strategy did not involve hunting or otherwise manipulating the experimental animals.

### 2.2 Sample collection

**Figure 1:**
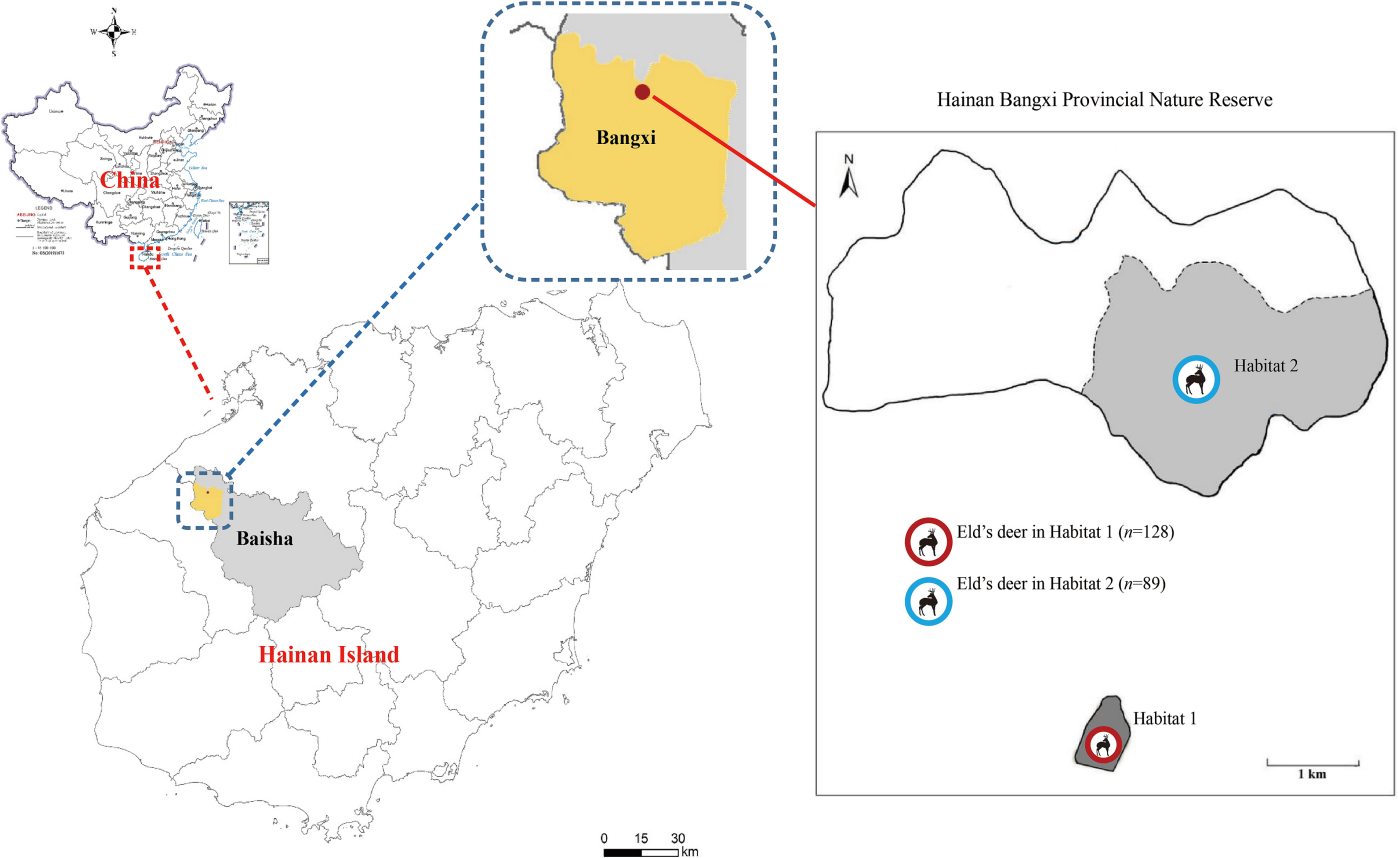
Distribution of sampling sites of Eld’s deer in the Hainan Bangxi Provincial Nature Reserve in the present study.

From March to August 2021, a total of 217 fresh fecal samples were collected from wild Eld’s deer in two completely isolated areas of Hainan Bangxi Provincial Nature Reserve: Habitat 1 (*n* = 89) and Habitat 2 (*n* = 128) (Figure 1). Fresh specimens (approximately 20 g) were immediately collected in sterilized 5-mL tubes with the assistance of experienced staff of the nature reserve, after observing the leaving of Eld’s deer. Each collected fecal sample should be kept more than 3 m apart to ensure that they were not from the same deer, and temporarily stored in a refrigerated insulated tank. All the samples were taken back to the laboratory for storage at −80°C until analysis.

### 2.3 DNA extraction and nested PCR amplification

Fecal samples were washed with distilled water and centrifuged at 1500×*g* for 10 min. This process was repeated three times. Genomic DNA was extracted directly from 200 mg of each processed fecal specimen using the QIAamp DNA stool mini kit (Qiagen, Hilden, Germany). The extraction procedure adhered to the manufacturer’s recommended protocol, with an elevated lysis temperature of 95°C to guarantee a high DNA yield. The extracted DNA was stored at −20°C until PCR analysis.

To assess the prevalence and genotypes of *E. bieneusi*, nested PCR assays were used to amplify a 390 bp fragment encompassing the ITS region as described in primers previously reported (20). Each PCR run included a positive control with DNA of the *E. bieneusi* BEB6 genotype from goat and a negative control (reagent-grade water without DNA). All the secondary PCR products were run on a 1.5% agarose gel and visualized by staining the gel with Goldenview.

### 2.4 Sequencing and phylogenetic analysis

Secondary PCR products of positive samples were sequenced in both directions using Big Dye Terminator v3.1 Cycle Sequencing Kit (Applied Biosystems, USA) and an ABI PRISM 3730 XL DNA Analyzer (Thermo Fisher Scientific, Waltham, MA, USA). Sequence accuracy was verified through bidirectional sequencing. The obtained nucleotide sequences were aligned with each other and compared to the reference sequences downloaded from GenBank using the Basic Local Alignment Search Tool (BLAST)^1^ and ClustalX 1.83^2^ in order to determine the genotypes. According to the established nomenclature system, the nucleotide sequences of the ITS region identical to known genotypes were given the first published name; the nucleotide sequences with single nucleotide substitutions, deletions, or insertions as compared to the known ITS genotypes were considered novel genotypes (21). Meanwhile, the novel genotypes were confirmed by sequencing another two separate PCR products of the same preparations.

A phylogenetic analysis was performed using the Neighbor-joining (NJ) method as implemented in MEGA 7,^3^ which was calculated by the Kimura 2-parameter model with 1,000 bootstrap replicates. The nucleotide sequences representative of the present study have been deposited in the GenBank database, with the corresponding accession numbers of OL603973 and OL603974 for *E. bieneusi*.

### 2.5 Statistical analysis

Statistical analysis were performed using Statistical Package for the Social Sciences (SPSS) version 22.0 (SPSS Inc., Chicago, IL, USA). Chi-square analysis was performed to compare the prevalence of *E. bieneusi* among different areas. The difference was considered statistically significant when the *p* < 0.05.

## 3 Results

### 3.1 Prevalence of *E. bieneusi*

The overall prevalence of *E. bieneusi* in Eld’s deer was 17.5% (38/217) in this study. Specifically, the infection rates were 13.5% (12/89) in Habitat 1, and 20.3% (26/128) in Habitat 2 (Table 1). There was no significant differences in infection rates between the two completely independent areas under investigation (*p* > 0.05).

**Table 1:** Prevalence and distribution of genotypes of *E. bieneusi* in Eld’s deer.

Location	Infection rate (%) (No. of positive/No. of examined)	Genotypes (*n*)
Habitat 1	13.5 (12/89)	Peru11 (10), HNED-I (1), HNED-II (1)
Habitat 2	20.3 (26/128)	D (19), EbpC (5), Peru8 (1), Type IV (1)
Total	17.5 (38/217)	D (19), Peru11 (10), EbpC (5), Peru8 (1), Type IV (1), HNED-I (1), HNED-II (1)

### 3.2 Characterization and distribution of *E. bieneusi* genotypes

**Figure 2:**
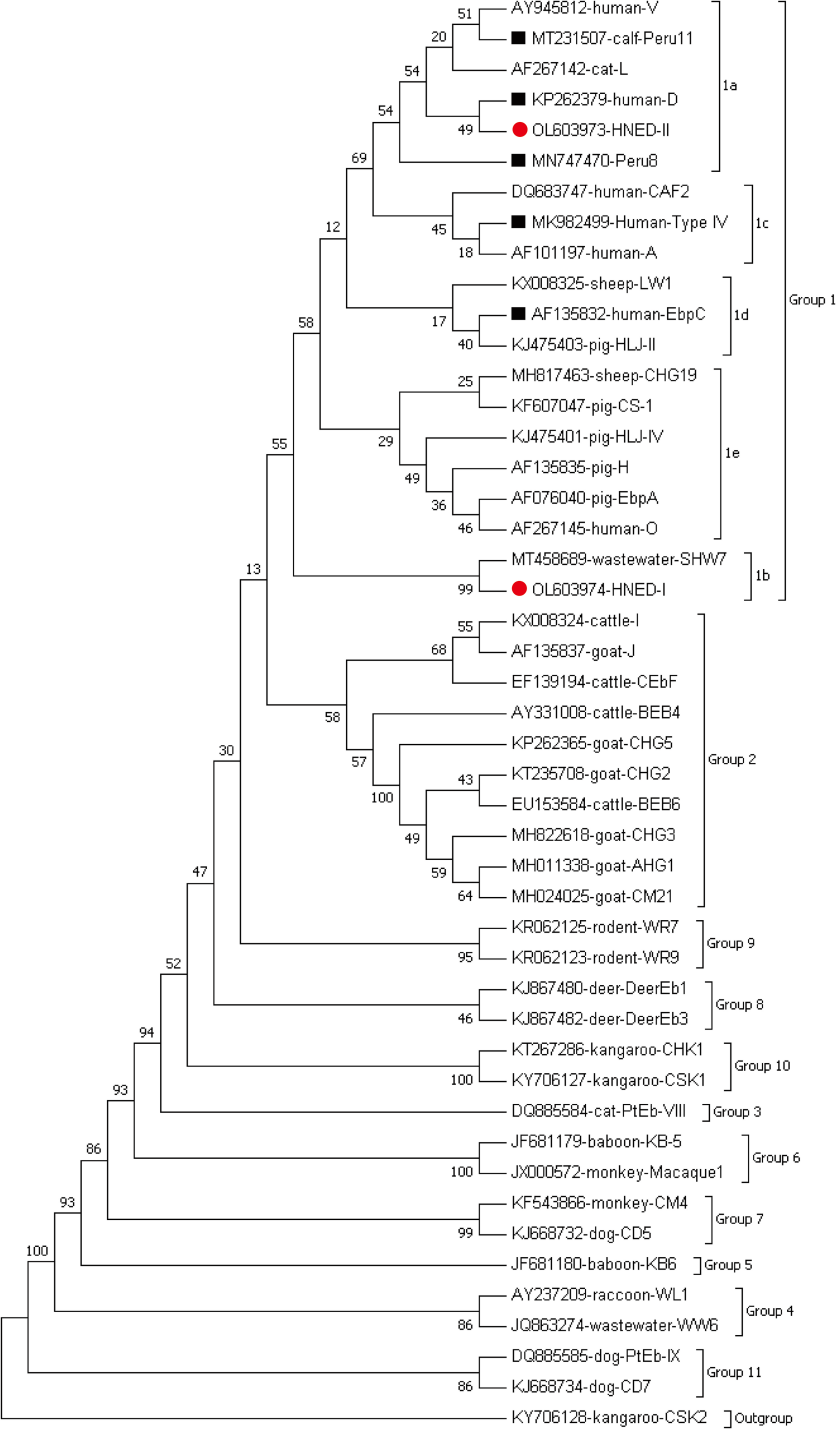
Phylogenetic relationships of representative sequences for the ITS genotypes of *E. bieneusi* identified from Eld’s deer in present study with reference sequences using maximum likelihood analysis. The known and novel genotypes identified in this study were indicated by black squares (■) and red circles(●), respectively. Genotype CSK2 from white kangaroo (KY706128) is used as the outgroup.

Seven genotypes were obtained from ITS sequencing of 38 *E. bieneusi* isolates, including five known genotypes: genotype D (*n* = 19), Peru 11 (*n* = 10), EbpC (*n* = 5), Peru 8 (*n* = 1) and Type IV (*n* = 1), and two novel genotypes: HNED-I (*n* = 1) and HNED-II (*n* = 1). Notably, the detected genotypes were different between two completely isolated habitats of Eld’s deer. The genotypes Peru 11, HNED-I and HNED-II were all detected in samples from Habitat 1, but the genotypes D, EbpC, Peru 8 and Type IV were all detected in samples from Habitat 2 (Table 1). The phylogenetic analysis of the ITS region of *E. bieneusi* divided the genotypes, which were identified in Eld’s deer in this study, all into Group 1 (Figure 2).

Among the 38 recognized sequences, two were novel and labeled as genotypes HNED-I (GenBank accession no: OL603974) and HNED-II (GenBank accession no: OL603973). Genotype HNED-I exhibited 97.53% similarity with genotype SHW7 (MT458689) from urban wastewater in China, and has four nucleotide substitutions at positions 128 (T → C), 198 (T → G), 218 (A → G) and 232 (C → G). Compared to genotype D (MN704918) from donkeys in China, genotype HNED-II exhibited 99.18% similarity and has two nucleotide substitutions at positions 3 (A → G) and positions 217 (G → A) (Table 2).

**Table 2:** Positions of nucleotide changes of known and novel genotypes of *E. bieneusi* isolates in present study.

Genotype	3	31	51	52	81	93	113	117	128	130	131	138	141	165	176	198	217	218	232	Accession no.	Type of genotypes
SHW7	A	A	G	T	T	T	T	T	T	G	A	A	T	T	G	T	G	A	C	MT458689	Reference
HNPL-I	·	·	·	·	·	·	·	·	C	·	·	·	·	·	·	G	·	G	G	OL603974	Novel
HNPL-II	G	G	·	·	C	C	C	·	·	·	G	G	·	G	A	·	A	·	·	OL603973	Novel
D	·	G	·	·	C	C	C	·	·	·	G	G	·	G	A	·	·	·	·	KP262379	Known
Peru11	·	G	·	·	C	C	C	·	·	A	G	G	·	G	A	·	·	·	·	MT231507	Known
Peru8	·	G	·	·	C	C	C	G	·	·	G	G	·	G	A	·	·	·	·	MN747470	Known
Type IV	·	G	·	·	C	·	C	G	·	·	G	G	·	G	A	·	·	·	·	MK982499	Known
EbpC	·	G	·	·	C	·	·	G	·	·	G	G	C	G	A	·	·	·	·	AF135832	Known

## 4 Discussion

To date, there have been near 20 reports on the molecular epidemiological research of *E. bieneusi* involving 13 cervid species worldwide, and the infection rates varied from 0 to 75.0% (Table 3). In present study, the overall prevalence of *E. bieneusi* in wild Eld’s deer in Hainan was 17.5%, which was higher than infection rate of captive Eld’s deer (14.3%) (22), sika deer (5.7–16.0%) (9, 22–24), red deer (6.8–8.3%), Siberian roe deer (11.1%) (25) and free-ranging Chinese water deer (7.5%) in China (23), wild red deer (1.5%) in Spain (26), Sambar deer (4.8%) in Australia (27) and white-tailed deer (12.2%) in the USA (28). However, it was considerably lower than the prevalence in captive hog deer (75.0%) (29), fallow deer (27.3%) (23), sika deer (28.6–44.1%), and red deer (20.0–37.5%) (29–31), free-ranging and wild Père David’s deer (24.5–35.2%) (23, 32–34) in China, wild Korean water deer (53.6%) in Korea (35), and white-tailed deer (32.5%) in the USA (36). Notably, the infection rate of *E. bieneusi* in wild Eld’s deer in this study not only was similar to those in wild reindeers (16.8%) (34) and captive sika deer (17.8%) in China (9), captive red deer (19.4%) in Spain (26), but also in the average rate of cervid species in China (19.3%) (37) and around the world (19.7%) (95% *CI*: 0.021–0.310, *I*^2^ = 97.651%, *p* = 0.001, Table 3). The different infection rates of *E. bieneusi* in cervids not only were significantly associated with deer species (23), but also were influenced by various living conditions, biogeographic distributions, age, susceptibilities and health status of individuals (9, 20, 29).

**Table 3:** Prevalence and distribution of genotypes of *E. bieneusi* in cervid species.

Species	Existence	Locations	Infection rate (%) (No. of positive/No. of examined)	Genotypes (n)	References
Chinese water deer (*Hydropotes inermis inermis*)	free-ranging	Beijing, China	7.5 (3/40)	HLJD-V (1), HND-I (1), BJCWD (1)	(23)
Fallow deer (*Dama dama*)	wild	Melbourne, Australia	0 (0/17)	—	(27)
	wild	BR1-5, Spain	0 (0/96)	—	(26)
	captive	Sichuan, China	0 (0/7)	—	(29)
	captive	Beijing, China	27.3 (15/55)	HLJD-V (2), BEB6 (2), MWC_d1 (1), BJFD (10)	(23)
Eld’s deer (*Rucervus eldii*)	wild	Hainan, China	17.5 (38/217)	D (19), Peru11 (10), EbpC (5), Peru8 (1), Type IV (1), HNED-I (1), HNED-II (1)	This study
	captive	Hainan, China	14.3 (1/7)	HNED-III (1)	(22)
Hog deer (*Axis porcinus*)	captive	Sichuan, China	75.0 (3/4)	BEB6 (2), CHS9 (1)	(29)
Korean water deer (*Hydropotes inermis argyropus*)	wild	Chungbuk, Jeonbuk, ChungNam, JeonNam and GyungNam, Korea	53.6 (52/97)	D (29), Korea-WL1 (12), Korea-WL2 (5), Korea-WL5 (1), Korea-WL6 (1)	(35)
Père David*’*s deer (*Elaphurus davidianus*)	wild	Henan, China	34.0 (16/47)	Type IV (4), EbpC (4), EbpA (4), BEB6 (2), COS-I (1), COS-II (1)	(32)
Père David*’*s deer (*Elaphurus davidianus*)	wild	Hubei, China	35.2 (45/128)	HLJD-V (42), MWC_d1 (3)	(33)
	free-ranging	Beijing, China	30.0 (24/80)	HLJD-V (12), MWC_d1 (4), BEB6 (1), BJED-I to BJED-V (7)	(23)
	free-ranging	Beijing, China	24.5 (70/286)	HLJD-V (35), MWC_d1 (14), BEB6 (3), D (2), Peru6 (1), BJED-I (2), BJED-II (5), BJED-III (2), BJED-IV (2), BJED-V (4)	(34)
Reindeers (*Rangifer tarandus*)	wild	Great Hinggan Mountains, China	16.8 (21/125)	CHN-RD1 (12), Peru6 (6), CHN-RD2 - CHN-RD4 (one each)	(49)
Red deer (*Cervus elaphus*)	wild	Melbourne, Australia	0 (0/77)	—	(27)
	wild	BR2 and BR3, Spain	1.5 (5/329)	EbCar2 (1), S5 (2), BEB17 (1), Type IV (1)	(26)
	captive	Heilongjiang, China	20.0 (1/5)	HLJD-V (1)	(30)
	captive	Heilongjiang, China	6.8 (3/44)	BEB6 (2), HLJD-VI (1)	(25)
	captive	Sichuan, China	25.0 (1/4)	BEB6 (1)	(29)
	captive	Liaoning, China	8.3 (5/60)	BEB6 (5)	(25)
	captive	Jilin, China	37.5 (6/16)	BEB6 (2), JLD-IV (3), JLD-XIII (1)	(31)
	captive	BR5, Spain	19.4 (63/324)	HLJD-V (43), BEB6 (3), MWC_d1 (1), Wildboar3 (6), DeerSpEb1 (7), DeerSpEb2 (13), DeerSpEb3 (1)	(26)
	wild	BR1-5, Spain	0 (0/93)	—	(29)
Sambar deer (*Rusa unicolor*)	wild	Melbourne, Australia	4.8 (25/516)	MWC_d1 (19), D (3), J (1), Type IV (1), MWC_d2 (1)	(27)
Siberian roe deer (*Capreolus pygargus*)	captive	Liaoning, China	11.1 (2/18)	BEB6 (2)	(25)
Sika deer (*Cervus nippon*)	captive	Jilin, China	44.1 (15/34)	BEB6 (12), HLJD-V (3)	(30)
	captive	Jilin, China	7.1 (23/326)	J (11), BEB6 (4), EbpC (1), CHN-DC1 (1), KIN-1 (1), JLD-1 (2), JLD-2 (2), JLD-3 (1)	(24)
	captive	Jilin and Henan, China	35.9 (215/599)	BEB6 (129), HLJDI (18), EbpC (3), HLJD-IV (2), COS-I (1), EbpA (1), D (1), JLD-I (7), JLD-II (5), HND-I (4), JLD-III (2), HND-II (1), JLD-IV (3), JLD-V (2), JLD-VI (5), HND-III (1), JLD-VII (1), JLD-VIII (16), JLD-IX (1), JLD-X (1), HND-IV (1), JLD-XI (2), JLD-XII (1), JLD-XIV (7)	(31)
	captive	Jilin, China	17.8 (96/538)	BEB6 (74), EbpC (3), I (1), JLD-III (1), JLD-IX (1), JLD-XV (2), JLD-XVI (1), JLD-XVII (2), JLD-XVIII (2), JLD-XIX (2), JLD-XX (2), JLD-XXI (2), JLD-XXII (1), JLD-XXIII (2)	(9)
	captive	Hainan, China	14.3 (1/7)	CM1 (1)	(22)
	captive	Heilongjiang, China	32.6 (13/52)	BEB6 (8), HLJD-I -HLJD-V (one each)	(30)
	captive	Heilongjiang, China	16.0 (13/81)	BEB6 (10), JLD-VIII (3)	(9)
Sika deer (*Cervus nippon*)	captive	Sichuan, China	28.6 (2/7)	BEB6 (1), SC03 (1)	(29)
	captive	Liaoning, China	5.7 (2/35)	LND-I (1), JLD-XVI (1)	(9)
	captive	Beijing, China	12.5 (5/40)	CGC2 (3), JLD-XV (2)	(23)
White-tailed deer (*Odocoileus virginianus*)	wild	New York, USA	12.2 (6/49)	WL18 (2), WL19 (2), WL4 (2)	(28)
	wild	Maryland, USA	32.5 (26/80)	WL4 (7), I (4), J (1), LW1 (1), DeerEb1-DeerEb13 (one each)	(36)
Total			19.7^a^ (816/4,540)	Group 1: HLJD-V (140), D (54), MWC_d1 (42), EbpC (16), Korea-WL1 (12), CHN-RD1 (12), BJFD (10), Peru11 (10), Type IV (7), Peru6 (7), JLD-I (7), DeerSpEb1 (7), Wildboar3 (6), JLD-II (6), HND-I (5), JLD-VI (5), Korea-WL2 (5), EbpA (5), JLD-III (3), S5 (2), JLD-2 (2), JLD-V (2), JLD-XVI (2), JLD-XVIII (2), JLD-XIX (2), WL18 (2), WL19 (2), Peru8 (1), HNED-I (1), HNED-II (1), Korea-WL5 (1), Korea-WL6 (1), EbCar2 (1), BEB17 (1), MWC_d2 (1), CHN-DC1 (1), KIN-1 (1), JLD-3 (1), JLD-XXII (1), HLJD-II (1), HLJD-III (1), SC03 (1), HND-II (1), HND-III (1), LW1 (1), CHN-RD2 - CHN-RD4 (oen each), DeerEb1- DeerEb13 (one each) Group 2: BEB6 (263), JLD-VIII (19), HLJDI (18), J (13), DeerSpEb2 (13), I (5), JLD-XIV (7), BJED-II (6), JLD-IV (6),	
Total			19.7^a^ (816/4,540)	BJED-V (5), JLD-XV (4), BJED-I (3), BJED-III (3), BJED-IV (3), HLJD-IV (3), CGC2 (3), COS-I (2), JLD-1 (2), JLD-XVII (2), JLD-IX (2), JLD-XX (2), JLD-XXI (2), JLD-XXIII (2), BJCWD (1), HNED-III (1), CHS9 (1), COS-II (1), HLJD-VI (1), JLD-XIII (1), DeerSpEb3 (1), JLD-VII (1), JLD-X (1), JLD-XI (1), JLD-XII (1), CM1 (1), HLJD-I (1), HND-IV (1), LND-I (1), Group 3: WL4 (9)	

a The random-effects model was used to analyse *E. bieneusi* infection in deer worldwide (95% CI: 0.021–0.310, Heterogeneity: *I*^2^ = 97.651%, *p* = 0.001).

At present, a total of 100 ITS genotypes of *E. bieneusi* with high genotypic heterogeneity and phenotypic diversity have been identified in cervid species, including 61 genotypes in Group 1, 38 genotypes in Group 2 and one in Group 3 (Table 3). Genotypes HLJD-V and BEB6 were the most popular genotypes in deer from China, and many other genotypes also have been detected in deer from Australia, Korea, Spain and the USA, such as D, MWC_d1, J, Korea-WL-, WL-, CHN- and JLD- associated genotypes. Many genotypes in Groups 1 and 2 have been previously discovered both in humans and animals, which implied that *E. bieneusi* might be spread from deer to humans (Table 3). In our research, 7 distinct genotypes were identified, including five known (D, EbpC, Peru11, Peru8 and Type IV) and two novel genotypes (HNED-I and HNED-II) (Table 1). All genotypes of were categorized into Group 1 (Figure 1). This result indicates a possible risk of zoonotic transmission, where these genotypes could potentially pass from Eld’s deer to humans. Genotype D was the most prevalent genotypes in Eld’s deer with the rate of 50.0% (19/38), which was similar to the results of previous studies on wild Korean water deer (35). Genotype D also were identified in wild Sambar deer in Australia (27), free-ranging Père David’s deer (*Elaphurus davidianus*) (34) and captive Sika deer (31) in China. Genotype D was known as the most prevalent zoonotic genotype and not only distributed in humans but also in livestock (sheep, goat, cattle, and pig), companion animals (cat and dog), wild animals (wild boar, wild deer, non-human primates, and tiger), and water sources worldwide (12). Genotypes Peru11, EbpC, Peru8 and Type IV have been frequently observed in humans and various animal hosts, including nonhuman primates, domesticated animals, and avian species (11, 38). To our knowledge, genotypes Peru11 and Peru8 have not been documented in deer previously. This work represented the initial detection of these two genotypes in cervid species, broadening their recognized range of hosts. Genotype EbpC has been detected in wild Père David’s deer (32) and captive Sika deer in China (9, 24, 31). Remarkably, genotypes Peru8 and EbpC have been reported in diarrheic livestocks, and genotype EbpC was the main genotype and demonstrating higher genetic diversity than others in diarrheic pigs in China (39–42), which implied that these 2 genotypes might be associated with intestinal disease in artiodactyl animals, including deer. Genotype Type IV was dominant genotype in wild Père David’s deer in Henan, China (32), which also was identified in wild Sambar deer in Australia (27) and Red deer in Spain (26). In our study, the novel genotype HNED-I showed the highest match (97.53% identity) with *E. bieneusi* genotype SHW7, obtained from urban wastewater in China in 2020 (43). Genotype SHW7 also has been found in civets and bamboo rats in Hainan (44, 45), and wild rats in Zhejiang, China (46). The novel genotype HNED-II exhibited 99.18% similarity with genotype D, obtained from donkeys in China in 2020 (47).

Despite no significant difference between infection rates of *E. bieneusi* in Eld’s deer from two completely isolated habitats, the ITS genotypes carried by Eld’s deer in perfectly independent habitats were rather different. Genotypes Peru11, HNED-I and HNED-II were detected in samples from Habitat 1, but genotypes D, EbpC, Peru8 and Type IV were identified from Habitat 2 in the nature reserve. Moreover, the genotype HNED-III was identified in captive Eld’s deer in Hainan Tropical Wildlife Park in our previous research (22). The similar results were found in research on *E. bieneusi* in Père David’s deer from Henan, Hubei and Beijing (23, 32, 33), and in giant pandas from Sichuan and Shaanxi in China (30, 48). These data suggest that the difference among genotypes of *E. bieneusi* in the same animal species may be related to living status, habitant environment and sources of infection. Currently, there were no reports on direct evidence of deer’s diarrhea caused by *E. bieneusi*, but it was crucial to persistently observe and comprehend the epidemiology of *E. bieneusi* in endangered Eld’s deer to acquire a more profound comprehension of its transmission patterns and prospective consequences on health and survival of Eld’s deer.

## 5 Conclusion

In summary, *E. bieneusi* infection was detected in wild globally endangered Eld’s deer for the first time. Seven ITS genotypes were identified and all belonging to zoonotic Group 1. The discovery of novel genotypes HNED-I and HNED-II offered more genetic diversity of *E. bieneusi*. Genotypes Peru11 and Peru8 were first identified in cervids in this study. The future studies should systematically focus on revealing the biological characteristics of *E. bieneusi* and assessing its potential threats to public health, veterinary, and Eld’s deer conservation.

## Data Availability

The datasets presented in this study can be found in online repositories. The names of the repository/repositories and accession number(s) can be found in the article/supplementary material.

## Footnotes

^1^ http://www.ncbi.nlm.nih.gov/BLAST/

^2^ http://www.clustal.org/

^3^ http://www.megasoftware.net/
